# Changes in United States Latino/a High School Students’ Science Motivational Beliefs: Within Group Differences Across Science Subjects, Gender, Immigrant Status, and Perceived Support

**DOI:** 10.3389/fpsyg.2019.00380

**Published:** 2019-02-22

**Authors:** Ta-yang Hsieh, Yangyang Liu, Sandra D. Simpkins

**Affiliations:** School of Education, University of California, Irvine, Irvine, CA, United States

**Keywords:** science, motivational beliefs, Latino, gender, immigrant generation status, science support, interest, ability self-concept

## Abstract

Science motivational beliefs are crucial for STEM (science, technology, engineering, and math) performance and persistence, but these beliefs typically decline during high school. We expanded the literature on adolescents’ science motivational beliefs by examining: (1) changes in motivational beliefs in three specific science subjects, (2) how gender, immigrant generation status, and perceived support from key social agents predicted differences in adolescents’ science motivational beliefs, and (3) these processes among Latino/as in the United States, whose underrepresentation in STEM is understudied. We used hierarchical linear modeling to estimate the changes in 104 (40% female) Latino/a high school students’ physics, chemistry, and biology motivational beliefs from 9th to 11th grade. Subject-specific ability self-concept, interest, and utility were regressed on gender, immigrant generation status, and perceived science support while controlling for family income, parent education, and adolescents’ school. Adolescents’ utility declined from 9th to 11th grade whereas their interest remained stable for all three science subjects. Adolescents’ ability self-concept increased for biology, decreased for physics, but remained stable for chemistry. Gender differences in adolescents’ motivational beliefs at 9th grade only emerged for physics utility as well as physics and chemistry interest; yet, there were no gender differences in how adolescents’ science motivational beliefs changed over time. Contrary to expectations, immigrant generation status was not significantly associated with adolescents’ science motivational beliefs at 9th grade or in terms of how they changed over time. Adolescents who perceived higher science support generally had higher motivational beliefs in 9th grade, but did not differ on their rate of change. Our findings highlight the need to examine specific science subjects, and that typical gender differences in adolescents’ motivational beliefs discussed in the literature may not generalize to all racial and ethnic groups.

## Introduction

Gender disparities in science have gained both research and policy interest in the United States. The most recent statistics show that although females have gained fair representation in the biological sciences, they are still significantly underrepresented in the physical sciences (National Science Foundation [NSF], 2017). The female underrepresentation in the physical sciences is even more pronounced for certain ethnic minorities such as Latino/as, who are the fastest growing underrepresented minority population in the United States. Latino/as account for 18% of the United States population and are projected to account for more than half of the school-aged Americans by 2050. Latino/as, however, only account for 5% of all computer/mathematical scientists and physical scientists in the United States (Fry and Gonzales, 2008; National Science Foundation [NSF], 2017). Most research to date focuses on ethnic group comparisons, which highlight the underrepresentation of Latino/as. It is also important to understand the differences *among* Latino/as as some Latino/as are succeeding and pursuing STEM (science, technology, engineering, and math) pathways. Studies focusing on a specific ethnic group can help determine the various developmental pathways for that group, interindividual differences in those processes, and what supports positive development. In other words, studies examining the heterogeneity *within* the Latino/a population that identify factors predicting favorable outcomes are needed. The aim of the current study, hence, was not to generalize findings to the entire population, but instead to provide an in-depth analysis on a specific ethnic group that is often underrepresented in the literature and in STEM fields.

The focal outcomes of the current study were Latino/a adolescents’ science motivational beliefs from 9th to 11th grade, which according to the expectancy-value theory are critical predictors of science achievement-related outcomes. Considering that high school is a time when United States students face life-altering decisions, such as attending college and selecting a major, motivational beliefs could significantly influence their pursuit into science majors or careers during this turning point (Sadler et al., 2012). Additionally, we examined both demographic (i.e., gender and immigrant generational status) and contextual factors (i.e., perceived science support from significant social agents) as predictors of those science motivational beliefs.

### Expectancy-Value Theory

According to the expectancy-value theory (Wigfield and Eccles, 2000), individuals will be more likely to choose, persist, and excel in a task or domain if they hold high motivational beliefs about that domain. With regard to science, people who have strong science motivational beliefs are more likely to pick science classes and college majors (Buday et al., 2012). Earning high grades and demonstrating achievement or mastery in science is not enough to support individuals’ continued pursuit of science. Individuals also need to value and believe they are good at science to select into and persist in science-related fields (Jacobs et al., 2005).

Three of the key motivational beliefs as described by the expectancy-value theory are interest, utility, and ability self-concept. Interest, in this case, is how enjoyable individuals find science to be. Utility pertains to how useful individuals think science is. Ability self-concept is how good individuals think they are in science. It is theorized that the more value people see in science and the more people expect themselves to excel, the more likely they would choose to engage and persist in science-related fields. Prior studies have indeed supported the positive associations between science motivational beliefs and favorable outcomes, such as higher achievement, engagement, and aspirations in science (Lau and Roeser, 2002; Singh et al., 2002; Osborne et al., 2003; Maltese and Tai, 2011; Nagengast and Marsh, 2012).

Individuals’ motivational beliefs are expected to change over time as they develop and gain more experience (Wigfield et al., 2015). Several studies consistently suggest that students’ academic intrinsic motivation, ability self-concept, and perceived value generally decline with age (e.g., Lepper and Henderlong, 2000; Gottfried et al., 2001; Fredricks and Eccles, 2002; Jacobs et al., 2002; Lepper et al., 2005; Wigfield and Wagner, 2005; Lazarides and Raufelder, 2017; Lazarides and Watt, 2017). However, changes in youth’s science motivational beliefs have received less attention and the existing findings are inconsistent. When science motivation was examined as science overall, white American adolescents’ science motivational beliefs declined over time (Gottfried et al., 2009). But, when science motivational beliefs were examined specifically within the physical sciences, most adolescents reported either stability or decrease in their ability self-concept and value (Wang et al., 2017). Although the Wang and colleague’s study (2017) is highly relevant to the current study, their participants were predominately white students. The science gaps and predictors vary across racial/ethnic groups (National Science Foundation [NSF], 2017) and the patterns found for white adolescents may not generalize to underrepresented groups (Hsieh and Simpkins, 2018). Thus, the first aim of the current study is to describe how Latino/a adolescents’ science motivational beliefs changed in high school.

Another limitation is that researchers have typically examined youth’s motivational beliefs in science overall without differentiating between the specific science subjects. Not only does the expectancy-value theory argue for the importance of domain-specificity, middle and high school students’ motivational beliefs differ based on the specific science subject. For example, middle school students, on average, were more interested in and placed higher value on biology compared with chemistry and physics (Bennett and Hogarth, 2009). Relatedly, high school girls reported stronger ability self-concepts in earth science than boys, whereas no gender differences were found in the physical and life sciences (Britner, 2008). Finally, both white and Latino/a 9th grade students held different ability self-concepts and values across biology, chemistry, and physics (Simpkins et al., 2015b). Indicated by these findings, collapsing biology, chemistry, and physics into ‘science’ could mask the differences youth see in these subjects and could instead provide an average that is not representative of their true beliefs about any particular science subject. Moreover, females are overrepresented in the biological sciences and underrepresented in the physical sciences, making collapsing multiple science subjects particularly problematic. To address these gaps in the literature, the current study examined the three major science subjects in United States high school curricula, namely biology, chemistry, and physics. In addition, we also took the average of the three subjects for the purpose of showing how overall ‘science’ might fail to represent the nuances that each subject offers.

The expectancy-value theory also posits that motivational beliefs are shaped by individual characteristics (e.g., gender), the cultural milieu and family demographics (e.g., immigrant generational status), and socialization by others (e.g., perceived support). In the following paragraphs, we reviewed the theory and literature on gender, immigrant generational status, and perceived support, respectively, as potential determinants of motivational beliefs.

### Gender Differences in the Trajectories of Students’ Motivational Beliefs

One of the most salient demographic factors that might influence students’ motivational beliefs in science is gender. According to the expectancy-value theory, gender stereotypes and expectations shape socializers’ behaviors and individuals’ personal and social identities, which influence their expectancies and the values they associate with specific domains. Indeed, female students tend to rate themselves lower on math and science motivational beliefs than male students (Stake and Nickens, 2005; Simpkins et al., 2006, 2015a,b; Kurtz-Costes et al., 2008; Sax et al., 2015; Lazarides and Watt, 2017). However, there is evidence that gender differences in students’ motivational beliefs vary across ethnic groups (Else-Quest et al., 2013; Hsieh and Simpkins, 2018). It is unclear if the typical gender differences found among white majority students will emerge between Latinas and Latinos. On one hand, traditional gender roles are a core Latino/a cultural value (Knight et al., 2010) and may amplify the stereotype favoring males in science and may more negatively influence females’ science motivational beliefs. On the other hand, Latinas often outperform Latinos in school, which suggest females’ motivational beliefs might be higher than males (Plunkett and Bámaca-Gómez, 2003). Only a couple studies to our knowledge addressed this question directly and showed that Latinos reported slightly higher science (general), biology, chemistry, and physics ability self-concepts than Latinas, but there was no gender difference in how much the adolescents valued science, biology, or chemistry (Else-Quest et al., 2013; Simpkins et al., 2015b).

Previous studies have predominately focused on mean-level differences between males’ and females’ science motivational beliefs at one time point; gender differences in the *changes* in students’ science motivational beliefs have largely been unexplored. The observation that females reported lower science motivational beliefs than males at end of high school could result from lower motivational beliefs for females at start of high school (9th grade), faster declines among females, or a combination of both. Longitudinal data would allow us to empirically address these possibilities. Findings regarding changes in United States students’ math motivational beliefs suggested a gendered rate of change where females’ math motivational beliefs declined slower than that of males and actually helped close the gender gap from kindergarten through high school (Fredricks and Eccles, 2002; Jacobs et al., 2002). The current study examined not only how gender predicted Latino/as’ science motivational beliefs at the beginning of high school, but also how gender predicted the rate of change of these beliefs over time. Given that females are underrepresented in physics and chemistry but overrepresented in biology (National Science Foundation [NSF], 2017), we expected Latinas’ physics and chemistry motivational beliefs would be lower at 9th grade and decline at a faster rate than Latinos’ beliefs. In contrast, we expected Latinas’ biology motivational beliefs to be higher at 9th grade and decline at a slower rate than Latinos’ beliefs.

### Immigrant Generational Status Differences in the Trajectories of Students’ Motivational Beliefs

In the expectancy-value theory, individuals’ motivational beliefs are shaped by the social and cultural contexts. An important indicator of Latino/a students’ social and cultural context and predictor of their academic achievement is immigrant generational status. Findings on the immigrant paradox in education suggests that third generation Latino/as (i.e., both parents and youth were born in the United States) underperform their first and second generation counterparts (i.e., parents were born outside of the United States) in school after controlling for family socioeconomic status (Palacios et al., 2008; García Coll et al., 2012; Greenman, 2013; Aretakis et al., 2015; Feliciano and Lanuza, 2017). This phenomenon is coined as paradoxical because despite potential language barriers and other burdens to adapt, Latino/a students who immigrated or whose parents immigrated outperform or show ‘super-achievement’ compared with youth who and whose parents were born in the United States (Harris et al., 2008). Possible mechanisms underlying the immigrant paradox include differences across generations in cultural identity and resources in the proximal community such that earlier generations of immigrants have more support from their community and identify more closely with their ethnic culture, both of which function as protective factors (Perreira et al., 2010; Aretakis et al., 2015). Although prior studies suggest that third generation Latino/as have lower academic achievement and attainment than their first and second generation counterparts, it has not been empirically shown whether students’ motivational beliefs follow the same trend. Taken together, immigrant generational status is a relevant predictor to examine, and it is hypothesized that third generation students would have lower science motivational beliefs in 9th grade and evidence faster declines over time than their first and second generation counterparts.

### Perceived Support as a Protective Factor

Unlike studies on ethnic minorities that tried to identify risk factors, we took a strength-based perspective and examined whether adolescents’ perceived level of science support from parents, teachers, friends, and siblings/cousins in 9th grade could help buffer them against the typical declines in students’ motivational beliefs. According to the expectancy-value theory, perceived support from key social agents is positively associated with adolescents’ motivational beliefs (Eccles et al., 1983). For high school students, parents, teachers, friends, and siblings jointly create the proximal social contexts for motivation development (Plunkett and Bámaca-Gómez, 2003; Alfaro and Umaña-Taylor, 2015; Lazarides et al., 2017). Perceived support from some or all of these social agents positively predicts adolescents’ concurrent motivational beliefs (Bouchey and Harter, 2005; Alfaro et al., 2006; Plunkett et al., 2008). Extending the previous literature, the current study examined how perceived science support from parents, teachers, friends, and siblings/cousins at the beginning of high school was associated with both concurrent levels of science motivational beliefs and *changes* in science motivational beliefs over the next 2 years. A relevant study by Alfaro and Umaña-Taylor (2015) showed that adolescents’ perceived support at the beginning of high school predicted their overall academic motivation in the same year, but not change over time. The current study is different from Alfaro and Umaña-Taylor (2015) in two ways. First, we examined science-specific support and motivational beliefs instead of academic support in general. Secondly, we included perceived support from friends, which Alfaro and Umaña-Taylor also argued to be salient particularly during adolescence but did not include in their study. We considered perceived support from parents, teacher, friends, and sibling to capture the fact the adolescents are embedded in and interact with multiple social agents.

It is important to examine support jointly as well as the independent roles of parents, teachers, siblings, and friends in predicting the trajectories of students’ motivational beliefs. Prior studies that examined support from specific social agents among predominantly white high school students showed positive associations between the students’ science motivational beliefs and their perceived support from parents, teachers, and friends (Adya and Kaiser, 2005; Leaper et al., 2012; Lazarides and Ittel, 2013; Rice et al., 2013). Additionally, most studies that focused on Latino/a adolescents also replicated such positive associations between adolescents’ overall academic or science motivational beliefs and their perceived support from their parents (Simpkins et al., 2015b), teachers, (Lewis et al., 2012; Riconscente, 2014), and siblings (Alfaro and Umaña-Taylor, 2010). Taken together, we examined adolescents’ perceived science support from parents, teacher, sibling, and friends not only jointly as a composite, but also (in follow up analyses) as individual predictors.

### Current Study

Based on both theory and empirical research, the current study examined changes in Latino/a high school students’ science motivational beliefs from 9th to 11th grade. We also took into account individual and contextual factors including gender, immigrant generation status, and perceived science support from key socializers. Specifically, three research questions were tested.

Research Question 1 asked how students’ science motivational beliefs changed from 9th to 11th grade. We first tested overall science across the three subjects and then examined biology, chemistry, and physics separately. Aligning with results from prior studies (e.g., Gottfried et al., 2009; Wang et al., 2017), we hypothesized that students’ motivational beliefs would decline over time.

Research Question 2 examined whether there were demographic differences in students’ motivational beliefs at 9th grade and in the changes over time. First, we examined gender differences. Given that physics and chemistry are stereotypically masculine subjects (Su et al., 2009; Sax et al., 2015), we expected females to have lower motivational beliefs in these two subjects at 9th grade and to evidence faster declines than males. In contrast, given that the biological sciences are now overrepresented by females (National Science Foundation [NSF], 2017), we expected females to have higher biology motivational beliefs at 9th grade and to evidence slower declines than males. Next, we examined whether there were differences based on students’ immigrant generation status. Specifically, we tested whether being third generation immigrant versus being first or second generation immigrant predicted differences in adolescents’ science motivational beliefs at 9th grade and how they changed from 9th to 11th grade. In accordance with the Latino/a immigrant paradox in education (Feliciano and Lanuza, 2017), we expected first and second generation immigrants to report higher 9th grade motivational beliefs and evidence slower declines compared with their third generation counterparts.

Lastly, Research Question 3 asked whether adolescents’ perceived science support in 9th grade predicted their concurrent science motivational beliefs and changes in those beliefs over time. With a strength-based perspective and supported by prior studies (e.g., Bouchey and Harter, 2005; Alfaro and Umaña-Taylor, 2015), perceived science support was hypothesized to serve as a buffer such that higher perceived science support would predict higher beliefs at 9th grade and slower declines over time. Research Questions 2 and 3 were tested in the same models with family income, parent education level, the adolescent’s school as covariates.

## Materials and Methods

### Sample

Participants of the current study were 104 Latino/a adolescents (40% female) from three high schools in Arizona, United States. When the data were collected, Arizona was one of the six states in the United States with a Latino/a population of two million or more (U. S. Census Bureau, Population Division, 2014). Just a few years prior to collecting these data, a new law (SB-1070) was debated and passed which allowed law enforcement to inquire about people’s immigration status. Scholars have documented the potential negative effects of the climate for the Latino/a ethnic community in Arizona, such as ethnic-based microaggressions impacting the daily experiences of adolescents and their families (e.g., Lin et al., 2016).

The participants were first recruited when they were in 9th grade (*M*_age_ = 14.50, *SD* = 0.52) during the 2012–2013 school year and were recontacted 1 and 2 years later (i.e., 10th and 11th grade). As part of a larger study, participants were recruited with one of their parents and an older sibling or cousin. Written consents/assents were obtained from all participants, and the study was approved by the Arizona State University institutional review board ethics committee. Data utilized in this study include adolescent-reported data at each time point and parent-reported data in 9th grade. Data collection in 9th and 10th grade mostly happened in participants’ homes with a few exceptions at the university or local library; data collection in 11th grade took place in adolescents’ schools. Adolescent participants and their parents separately completed questionnaires with trained, bilingual Latino/a interviewers. All questionnaires were available in both Spanish and English. All but one student completed their surveys in English; 43% of parents completed the survey in English. Surveys were translated by bilingual research assistants and checked for accuracy using a forward-translation and panel method approach (Knight et al., 2009). Adolescents and parents each received $50 as compensation each year.

In terms of academics, 9% of the adolescents took honors science classes in 9th grade. The majority of the adolescents were either first or second generation (61%) and Mexican-origin^1^ (82%). Around 62% of the adolescents came from 2-parent, married families and 4.8% of parents had a science-related job that required a college degree. Average maternal education was high school. The median household income in 9th grade was $40,000–$49,000.

### Measures

In 9th, 10th, and 11th grade, students self-reported three motivational beliefs in biology, chemistry, and physics. Ability self-concept was measured using four items (e.g., ‘how good at [biology/chemistry/physics] are you?’; α = 0.89–0.93). Interest was measured with two items [e.g., ‘how much do you like [biology/chemistry/physics]?’; Spearman’s rho = 0.74–0.88 (*p* < 0.001)] and utility was measured with three items (e.g., ‘how useful is what you learn in [biology/chemistry/physics]?’; α = 0.88–0.94). All nine measures (i.e., three motivational beliefs by three subjects) were on 7-point Likert scales. The scales were used in prior studies in accordance to the expectancy-value theory (e.g., Simpkins et al., 2015a). These scales are invariant across Latina, Latino, white male, and white female high school students (Simpkins et al., 2015b). See Supplementary Table S1 for the list of all motivational beliefs items.

Perceived science support was a composite of 15 items (see Supplementary Table S1 for a list of all items), each asked in regard to support provided by their parents, older sibling or cousin, science teacher, and same-grade school friends on a 5-point scale (1 = *never*, 5 = *always*). The scale has been used in prior studies (Bouchey and Harter, 2005; Simpkins et al., 2015b, 2018). For each of the 15 items, participants’ responses were recoded to 1 if they rated “sometimes” (i.e., the mid-point of the scale) or higher from at least one of the four social agents. That is, participants’ responses were recoded to 0 if they did not rate “sometimes” or more from any of the four social agents. The 15 items were then summed to create a scale of perceived science support ranging from 0 to 15, with higher scores indicating more perceived support from at least one of their social agents (α = 0.89). We also conducted a series of follow up analyses examining perceived science support from each *specific* social agent, for which participants’ responses on each item were also recoded using the “sometimes” cutoff into 0 and 1. The 15 items were then summed within each social agent, yielding four scales that all ranged from 0 to 15.

A range of demographic variables were included in the current study. Adolescents reported their gender and whether they were born in the United States. Parents also reported their own birth country, family income (categorically on the scale of $10,000; 0 = “*less than $10,000*”, 10 = “*over 100,000*”), and level of education (six categories ranging from less than high school to professional degree). Adolescents were coded as third generation if they and at least one of their parents was born in the United States, otherwise adolescents were coded as first or second generation. Two dummy variables were generated to control for the three schools that participating adolescents attended in 9th grade.

### Plan of Analysis

Among the 104 high school students recruited in the study, 86 had complete data on all focal variables from 9th to 11th grade. We examined if students with and without complete data across the three time points differed on family income, maternal education, gender, immigrant generational status, 9th grade science grade, 9th grade science motivational beliefs, and 9th grade perceived support. Results indicated that there were no significant differences between the two groups on family income [*t*(101) = -0.24, *p* = 0.81, Cohen’s *d* = 0.06], gender ([*X*^2^(1)] = 0.15, Cramer’s *V* = 0.04), immigrant generational status ([*X*^2^(1)] = 0.19, Cramer’s *V* = 0.04), 9th grade science grade [*t*(101) = 0.13, *p* = 0.89, Cohen’s *d* = 0.02], and overall perceived support [*t*(102) = 0.43, *p* = 0.66, Cohen’s *d* = 0.12]. There was a small difference in maternal education [*t*(98) = -1.13, *p* = 0.26, Cohen’s *d* = 0.30], and in two of the nine motivational beliefs. Specifically, adolescents with complete data across the 3 years had higher value of physics utility [*t*(100) = 2.66, *p* = 0.01, Cohen’s *d* = 0.68] and were more interested in chemistry at 9th grade [*t*(98) = 2.46, *p* = 0.02, Cohen’s *d* = 0.63] than adolescents with missing data.

Research Question 1 tested how science motivational beliefs changed from 9th to 11th grade in HLM version 7.3 (Raudenbush et al., 2017). Twelve linear growth curve models (i.e., time nested within person) were estimated for each of the three motivational beliefs (i.e., ability self-concept, interest, and utility) in science overall and separately in each of the three specific science subjects (i.e., biology, chemistry, and physics).

Research Questions 2 and 3 examined whether students’ science motivational beliefs differed by gender, immigrant generation status, and perceived science support, respectively. Gender, immigrant generation status, and perceived science support were added as predictors of the intercept and slope that evidenced significant variance from the HLMs described under Research Question 1. A significant predictor of the intercept suggests that adolescents’ motivational beliefs at 9th grade differed based on that predictor, whereas a significant predictor of the slope suggests that the changes in students’ motivational beliefs differed based on that predictor. Family income level, parent education level, and the school that the adolescents attended were added as covariates.

## Results

Table 1 and Supplementary Table S2 presents the descriptive statistics and bivariate correlations of all variables used in this study.

**Table 1:** Descriptive statistics.

		9th grade		10th grade		11th grade	
		Mean	*SD*	Mean	*SD*	Mean	*SD*
Biology							
	Ability self-concept	4.22	1.10	4.76	0.95	4.66	1.05
	Interest	4.81	1.32	4.91	1.42	4.97	1.28
	Utility	4.98	1.17	4.91	1.16	4.67	1.25
Chemistry							
	Ability self-concept	4.25	1.05	4.26	1.00	4.18	1.18
	Interest	4.41	1.34	4.35	1.43	4.41	1.49
	Utility	4.69	1.13	4.50	1.38	4.47	1.39
Physics							
	Ability self-concept	4.38	1.14	4.19	1.03	4.03	0.95
	Interest	4.56	1.31	4.38	1.23	4.39	1.23
	Utility	4.79	1.15	4.60	1.32	4.42	1.30
Predictors							
	Female	0.40	0.49				
	Third generation	0.39	0.49				
	Perceived science support	12.69	3.29				


*SD, standard deviation.*

### Changes in Adolescents’ Science Motivational Beliefs

**FIGURE 1:**
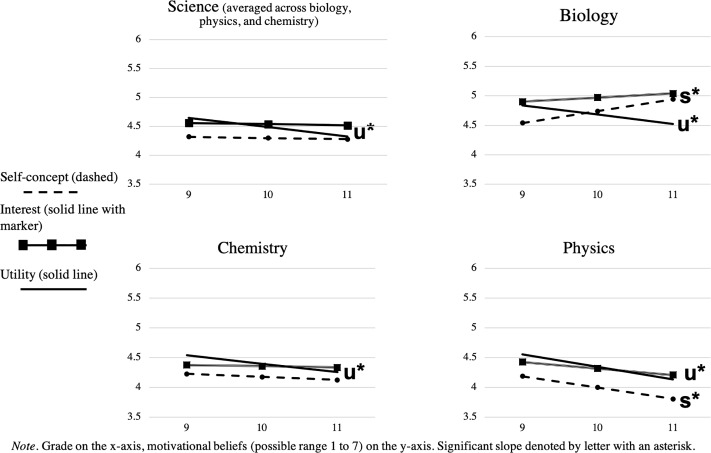
Changes in Science, Biology, Chemistry, and Physics Motivational Beliefs. Grade on the *x*-axis, motivational beliefs (possible range 1–7) on the *y*-axis. Significant slope denoted by letter with an asterisk.

Results from hierarchical linear models are presented in Figure 1 and Table 2. Results suggest that the mean of adolescents’ 9th grade motivational beliefs fell between 4.19 and 4.90, which corresponded to just above the mid-point on our 7-point Likert scale. The mean of the slope indicates the change in adolescents’ motivational beliefs where significant positive values suggest increases, significant negative values suggest decreases, and non-significant values suggest stability (i.e., no change). The findings for utility value and interest were consistent across the three science subjects though the patterns of change varied across the two types of beliefs. Adolescents’ utility values decreased for all science subjects from 9th to 11th grade (β = -0.16, -0.16, -0.14, -0.21, respectively, for science, biology, chemistry, and physics, *p* < 0.05). In contrast, adolescents’ interest remained stable for all science subjects (β = -0.02, 0.07, -0.02, -0.11, *ns*). The patterns for adolescents’ ability self-concepts varied by science subject. Adolescents’ ability self-concepts in science overall remained stable over time (β = -0.02, *ns*). However, adolescents’ ability self-concept decreased for physics (β = -0.19, *p* < 0.01), remained stable for chemistry (β = -0.05, *ns*), and increased for biology (β = 0.20, *p* < 0.001). Our results showed that if we simply grouped physics, chemistry, and biology together as ‘science,’ the opposing changes in adolescents’ ability self-concept by subject would have gone undetected.

**Table 2:** Estimates from hierarchical linear models.

		Mean at 9th grade (intercept)	Intercept variance	Change from 9th to 11th grade (slope)	Slope variance
Science					
	Ability self-concept	4.32 (0.07)^∗∗∗^	0.49^∗∗∗^	–0.02 (0.05)	0.11^∗∗∗^
	Interest	4.56 (0.10)^∗∗∗^	0.79^∗∗∗^	–0.02 (0.05)	0.09^∗∗∗^
	Utility	4.65 (0.10)^∗∗∗^	0.90^∗∗∗^	–0.16 (0.05)^∗∗^	0.09^∗∗∗^
Biology					
	Ability self-concept	4.54 (0.08)^∗∗∗^	0.39^∗∗∗^	0.20 (0.06)^∗∗∗^	0.02
	Interest	4.90 (0.11)^∗∗∗^	0.97^∗∗∗^	0.07 (0.07)	0.16^∗∗∗^
	Utility	4.84 (0.10)^∗∗∗^	0.84^∗∗∗^	–0.16 (0.06)^∗^	0.18^∗∗∗^
Chemistry					
	Ability self-concept	4.23 (0.08)^∗∗∗^	0.52^∗∗∗^	–0.05 (0.07)	0.24^∗∗∗^
	Interest	4.38 (0.11)^∗∗∗^	0.91^∗∗∗^	–0.02 (0.08)	0.13
	Utility	4.54 (0.11)^∗∗∗^	1.05^∗∗∗^	–0.14 (0.07)^∗^	0.14^∗∗^
Physics					
	Ability self-concept	4.19 (.09) ^∗∗∗^	0.65 ^∗∗∗^	–0.19 (0.05)^∗∗∗^	0.09^∗∗^
	Interest	4.43 (.10) ^∗∗∗^	0.91 ^∗∗∗^	–0.11 (0.07)	0.19^∗∗∗^
	Utility	4.56 (.11) ^∗∗∗^	1.10 ^∗∗∗^	–0.21 (0.06) ^∗∗∗^	0.12^∗∗∗^


*Coefficients are unstandardized. Science refers to the average of biology, chemistry, and physics. ^∗^p < 0.05, ^∗∗^p < 0.01, ^∗∗∗^p < 0.001.*

Variances of intercept and slope denote if there were interindividual differences among adolescents in terms of their motivational beliefs at 9th grade and in the changes of their motivational beliefs over time (Table 2). There were significant interindividual differences in adolescents’ motivational beliefs in all science subjects in 9th grade and in the rate of change from 9th to 11th grade in most subjects. The variance of the slope was not statistically significant for biology ability self-concepts and chemistry interest, suggesting that adolescents did not differ significantly from each other in the rate of change on these two beliefs. Biology ability self-concept and chemistry interest, therefore, were not included in the subsequent predictive analyses. Although some slopes had non-significant means (e.g., chemistry ability self-concept, biology interest, and physics interest), meaning that the adolescents *on average* did not increase or decrease in those motivational beliefs over time, that should not be confused with the presence of significant *variance* in slope, which means that there were significant differences *between adolescents* in their change in the motivational beliefs.

### Predictors of Students’ Motivational Beliefs at 9th Grade and Change Over Time

We tested gender, immigrant generational status, and perceived science support as predictors to examine differences among Latino/a students in the trajectories of their science motivational beliefs (Table 3). Contrary to our expectations, our findings suggested minimal gender differences in students’ science motivational beliefs at 9th grade and changes over time. Latinas reported lower physics interest and utility (β = -0.62, -0.49, *p* < 0.05) in addition to lower chemistry interest (β = -0.62, *p* < 0.01) than Latinos at the start of high school (i.e., 9th grade). There were no gender differences at 9th grade for the other motivational beliefs (β ranged -0.30 to 0.17, *ns*). Latinas also did not differ from Latinos in how much their science motivational beliefs changed from 9th to 11th grade (β ranged -0.25 to 0.02, *ns*). Although most of the coefficients were negative, meaning that Latinas on average reported faster declines than Latinos, those differences were not statistically significant.

**Table 3:** Gender and perceived support predicting changes in science motivational beliefs.

		Science			Biology			Chemistry			Physics		
		Ability self-concept	Interest	Utility	Ability self-concept	Interest	Utility	Ability self-concept	Interest	Utility	Ability self-concept	Interest	Utility
Female	On intercept (*SE*)	–0.17 (0.15)	–0.46 (0.19)^∗^	–0.20 (0.20)	0.06 (0.17)	–0.14 (0.23)	0.17 (0.20)	–0.29 (0.17)	–0.62 (0.23)^∗∗^	–0.30 (0.22)	–0.23 (0.17)	–0.63 (0.21)^∗∗^	–0.49 (0.22)^∗^
	On slope (*SE*)	–0.11 (0.09)	–0.15 (0.11)	0.03 (0.11)	–	–0.21 (0.16)	–0.05 (0.13)	–0.09 (0.14)	–	0.15 (0.15)	–0.07 (0.11)	–0.24 (0.15)	0.02 (0.12)
Perceived support	On intercept (*SE*)	0.04 (0.02)^∗^	0.07 (0.03)^∗∗^	0.10 (0.02)^∗∗∗^	0.05 (0.02)^∗∗^	0.08 (0.03)^∗∗^	0.10 (0.02)^∗∗∗^	0.03 (0.02)	0.05 (0.03)	0.08 (0.03)^∗∗^	0.04 (0.02)	0.07 (0.03)^∗∗^	0.10 (0.03)^∗∗∗^
	On slope (*SE*)	–0.01 (0.01)	–0.02 (0.01)	–0.00 (0.01)	–	–0.03 (0.02)	–0.01 (0.02)	–0.01 (0.02)	–	0.00 (0.02)	–0.02 (0.01)	–0.02 (0.02)	0.00 (0.02)


*School (2 dummy variables), family income, and parent educational level were included as covariates. Immigrant generation status was also included as predictor, but the results are not shown. SE, standard error. – means insignificant variance to predict interindividual differences. ^∗^*p* < 0.05, ^∗∗^*p* < 0.01, ^∗∗∗^*p* < 0.001.*

We expected third generation Latino/as would report lower science motivational beliefs in 9th grade and faster declines than first and second generation Latino/as. However, those differences were not statistically significant. Third generation students did not differ on the level of their science motivational beliefs at 9th grade (β ranged -0.21 to 0.50, *ns*), nor did they differ on the changes in these beliefs from 9th to 11th grade compared with first and second generation students (β ranged -0.27 to 0.18, *ns*).

We expected adolescents who perceived higher science support to have higher science motivational beliefs at 9th grade and evidence slower declines over the next 2 years. When support from multiple social agents was examined as a composite, adolescents’ perceived science support at 9th grade positively predicted concurrent science motivational beliefs, but not the changes in their beliefs over time. Adolescents who perceived higher science support from key social agents in 9th grade also had higher motivational beliefs for science overall and for all three science subjects (β ranged 0.04 to 0.10, *p* < 0.05), except chemistry interest (β = 0.05, *p* = 0.06) as well as chemistry and physics ability self-concept (β = 0.03, 0.04, *ns*; Table 3). Adolescents’ perceived science support in 9th grade, however, did not predict rate of change in any of the motivational beliefs tested (β ranged -0.03 to 0.01, *ns*).

In addition to examining perceived science support as a composite across the four social agents, we conducted follow up analyses to investigate the association between perceived support from each social agent and students’ science motivational beliefs. Specifically, perceived science support from parents, teachers, friends, and siblings/cousins were analyzed in separate models to predict adolescents’ science motivational beliefs while holding the same set of demographic covariates constant. Results (Supplementary Table S3) largely aligned with that when perceived science support was operationalized as a composite of the four social agents. Specifically, most science motivational beliefs in 9th grade were positively associated with concurrent perceived science support from parents (β ranged 0.05 to 0.07, *p* < 0.05; except ability self-concept and interest in chemistry and physics), siblings/cousins (β ranged 0.03 to 0.08, *p* < 0.05; except physics ability self-concept and interest in biology and physics), and friends (β ranged 0.04 to 0.07, *p* < 0.05). Perceived science support from teachers positively predicted utility in all three science subjects (β = 0.07, *p* < 0.05) and biology ability self-concept (β = 0.04, *p* < 0.01). Also aligning with results when perceived science support was operationalized as composite of the four social agents, how much the adolescents’ science motivational beliefs changed over time was largely not predicted by perceived science support from parents, siblings/cousin, friends, or teachers. The only exceptions were that higher perceived support from parents and friends predicted faster declines in adolescents’ physics ability self-concept, and that higher perceived support from teacher predicted faster decline in biology interest.

## Discussion

Students’ motivational beliefs are malleable and change over time. Thus, it is essential to examine them with a developmental perspective instead of in a snapshot (i.e., single timepoint measurement). The current study examined the stability and change in United States Latino/a adolescents’ science motivational beliefs during the first 3 years of high school. Results suggested that students’ utility declined for chemistry, physics, and biology, whereas interest in all three subjects remained stable. Students’ ability self-concepts decreased for physics, remained stable for chemistry, and increased for biology from 9th to 11th grade. Our findings mostly align with prior work that found decline and stability in adolescents’ motivational beliefs in physical sciences (Wang et al., 2017), and we expand the literature by including biology. These findings highlight the need to examine specific science subjects and specific science motivational beliefs. Combining physics, chemistry, and biology simply as science would have masked the divergent changes in students’ ability self-concepts over time. In addition to differentiating between subjects within the sciences, our study also differentiated motivational beliefs in terms of interest, utility value, and ability self-concept. Although these motivational beliefs are often correlated with one another, they are theoretically distinct and serve different functions (Wigfield et al., 2015).

The minimal gender differences in the changes of motivational beliefs was unexpected given the gender differences found in prior work (e.g., Simpkins et al., 2015b). In the current study, Latinos reported higher motivational beliefs at the beginning of high school than Latinas only for chemistry interest, physics interest, and physics utility, but not for the other motivational beliefs. Our findings aligned better with Hyde’s gender similarities hypothesis that demonstrated more within-gender than between-gender differences regarding science and math (Hyde and Linn, 2006). The gender similarities hypothesis points out that although gender difference might occur at the mean level, the female and male *distributions* of science and math achievement overlap more than they differ (Hyde, 2005). For example, the patterns of United States high school student’s math motivational beliefs showed no less difference within gender (by race/ethnicities) than between gender (Hsieh and Simpkins, 2018). The same study also showed that gender differences in math motivational beliefs among white students did not replicate for ethnic minority students such as Latino/as.

Another unexpected finding was that immigrant generation status did not predict students’ science motivational beliefs at 9th grade or the changes over time. Prior studies have pointed out that the immigrant paradox is more likely to emerge under certain conditions. For example, the paradox is more pronounced in schools with a more negative school climate, such that, third generation ethnic minority youth are more susceptible to negative peer influences (Greenman, 2013). Because positive school climate supports high school student’s motivational beliefs (Fan and Williams, 2018), a future direction is to bridge these two bodies of literature and examine whether differences by immigrant generation status emerge for the association between school climate and adolescent’s motivational beliefs.

Lastly, in regard to perceived science support, we found that students who perceived higher science support in 9th grade also tended to report higher science motivational beliefs at the same time. This finding aligns with theory and the body of literature suggesting that support from parents, teachers, friends, siblings, and other significant social agents are assets for Latino/a students’ educational trajectories (Bouchey and Harter, 2005; Plunkett et al., 2008). Students’ perceived science support at 9th grade, however, did not consistently predict *changes* in their science motivational beliefs from 9th to 11th grade. Our finding that perceived science support at 9th grade predicted initial level but not change in science motivational beliefs aligned with Alfaro and Umaña-Taylor (2015) findings on general academic support and motivational beliefs. Perhaps it is not enough to only look at support at one time point. It might be the case that changes in support over time would predict changes in motivational beliefs. However, we are limited by the sample size to explore this possibility. We did a series of follow up analyses to examine the associations between adolescents’ science motivational beliefs and their perceived science support from each of the social agents separately. Those results largely aligned with the conclusions when perceived science support was operationalized as a composite from the four social agents.

### Potential Implications

Overall, the results of the current study have potential research and practical implications. In terms of research implications, we provided empirical evidence for the need to distinguish among students’ motivational beliefs (i.e., ability self-concept, interest, and utility) and among specific science subjects (i.e., chemistry, physics, and biology). Additionally, we tested the expectancy-value theory within an often understudied ethnic population and showed that some, but not all, of the hypothesized mechanisms emerged for our Latino/a sample. Therefore, scholars should continue to interrogate when expected patterns generalize to other groups and under what circumstances they do not generalize and why that might be.

In terms of practical implications, our results suggest that the importance Latino/a high school students attach to biology, chemistry, and physics declines from 9th to 11th grade as does their beliefs about their physics abilities. These patterns mostly emerged regardless of adolescents’ gender and immigrant generation status. We envision two immediate implications for schools. First, schools should consider how to bolster the importance students attribute to these areas of science. Harackiewicz et al. (2012) bolstered parents’ and high school students’ value of science by providing informational resources which had real impacts on the courses students took and their subsequent college majors. Second, the lack of differences based on adolescents’ gender and immigration generation challenges prevalent stereotypes of who pursues science (National Science Foundation [NSF], 2017). Though it is possible that our analyses were underpowered to detect these differences and our findings need to be replicated, it is also possible that the typical patterns might not hold true for a specific and often understudied subgroups (e.g., Hsieh and Simpkins, 2018). These divergent findings are a cautionary message for both schools and researchers. Schools are likely to be more effective if they tailor to the lived experiences of their students in their specific communities instead of relying on the aggregated experiences of students from the national level. Relatedly, more *within-*group studies are needed before we use findings on the average population to problematize a specific ethnic, racial, immigrant, or gender groups and deepen stereotypical images of them.

Adolescents’ perceived science support from parents, siblings/cousins, teachers, and friends was positively related to their concurrent science motivational beliefs at the beginning of high school– highlighting the importance of encouraging support from those social agents. Though people may experience challenges in supporting adolescents if they did not take the same subjects or if adolescents’ schooling surpasses that of the person providing the support, it is important to note that there are many ways people can be supportive. Our measure of perceived support includes some aspects that do not necessarily require much prior knowledge of science (e.g., help you feel better when science is hard). Thus, parents and other social agents who have varying levels of science knowledge and educational capital can be empowered to serve as positive social agents. Although the socio-political context often poses barriers for Latino/a adolescents and their families (e.g., Lin et al., 2016), our study showed with a strength-based perspective that they have the potential to succeed with support from the people around them.

### Limitations and Future Directions

Although one strength of the current study is its longitudinal nature, studies spanning longer time frames would provide a more complete description of development. The current study speaks to high school, which has been shown to be a crucial developmental period in regard to STEM pathways and science motivational beliefs (Sadler et al., 2012), but future studies could expand to include earlier and subsequent developmental periods (e.g., Jacobs et al., 2002; Robinson and Lubienski, 2011) and crucial *transitional periods* such as the transition from middle to high school or the post high school transition to college or work.

Although our within-group focus is a strength and extends the current literature, the Latino/a adolescents in our study were diverse on some indicators but more homogeneous on other indicators (e.g., United States region). We examined heterogeneity among the participants in terms of gender, immigrant generational status, and perceived science support. Future studies could incorporate other factors that are relevant to the Latino/a population in the United States, such as socioeconomic status, country of origin, which United States region they live in, language ability, sense of family obligation, school ethnic composition, legal status, or the political climate (Goldsmith, 2004; Suárez-Orozco and Carhill, 2008; Greenman and Hall, 2013). For example, although prior studies mostly showed positive association between socioeconomic status and academic achievement for the Latino/a population (Altschul, 2012), the association with motivational beliefs might not be as strong (St-Hilaire, 2002) and associations between socioeconomic status and motivational beliefs of *specific science subjects* remain underexplored. Another example is that Latino/a youth’s educational experiences differ depending on where they live in the United States. While Latino/a youth in regions with a smaller Latino/a population and new receiving communities tend to face more discrimination than those in minority-majority regions, their identification with the local Latino/a community could promote their academic motivational beliefs (Perreira et al., 2010). Taken together, future studies could build on ours by examining other mechanisms that contribute to the diversity among Latino/as in the United States. The current sample size was modest and might have rendered some of our analyses under-powered. Studies that address a variety of indicators will require larger sample sizes than the current one to have adequate power to detect differences among multiple indicators of within-group heterogeneity.

We found that perceived science support positively predicted some aspects of science motivational beliefs when support was operationalized as the joint support from multiple social agents. The follow up analyses that examined perceived science support from each social agent in separate models also mostly point to the same conclusion. We presented the former as main analyses and the latter as follow up because we believe perceived science support as jointly from multiple social agents better reflect the experiences of adolescents as they are simultaneously interacting with the multiple social agents. To expand on the conceptualization of perceived science support, future studies could go into the nuances by differentiating the *patterns* of support from multiple social agents (Furrer and Skinner, 2003; Simpkins et al., 2018). Relatedly, perceived science support as operationalized by the current study consisted of both instructional support (e.g., help enroll the adolescent in science lessons, workshops, or tutoring programs outside of class) and motivational support (e.g., help the adolescent feel better when science is hard), but future studies could examine whether one kind of support is more predictive of science motivational beliefs than the other. Lastly, although we distinguished motivational beliefs by specific science subjects (i.e., chemistry, physics, and biology), our measure of perceived science support focused on science instead of specific science subject. This could have weakened the relations between perceived support and adolescents’ motivational beliefs, particularly when considering changes over time as students take different science subjects over time. People’s support may vary based on the science subject.

Also regarding our measures, the first two waves of data collection largely happened in adolescents’ homes whereas the last wave of data collection happened in their school. Though the means for the 11th grade motivational beliefs compared to 9th or 10th grade motivational beliefs do not suggest there was a large impact of context on the data, the change in context is confounded with the adolescents progressing from 10th to 11th grade. Theoretically, the context in which data are collected could impact students in positive or negative ways depending on the climate of their home and school contexts. Future studies could examine the impact of context by comparing the same data collected in various natural or experimental contexts.

## Conclusion

For our sample of 104 Latino/a adolescents, their motivational beliefs (i.e., interest, utility, and ability self-concept) in physics, chemistry, and biology either decreased or remained stable from 9th to 11th grade, except the increases in their biology ability self-concept. Our findings highlighted the need to differentiate both among science subjects and among motivational beliefs. Adolescents’ science motivational beliefs at 9th grade and the changes in those beliefs over time largely did not differ by gender or generation status. Adolescents’ perceived science support explained some differences in their science motivational beliefs at 9th grade. Overall, our study contributes to the literature by examining subject-specific motivational beliefs and within-group differences of an often understudied ethnic group. Additionally, we incorporated both demographic (i.e., gender, generation status) and contextual factors (i.e., perceived science support) that are theorized to be salient determinants of students’ science motivational beliefs.

## Author Contributions

T-yH, YL, and SS contributed to conceptualizing the study. T-yH wrote the first draft. YL and SS co-authored this study and helped to revise all drafts. SS conceived of the study from which the current data were drawn.

## Conflict of Interest Statement

The authors declare that the research was conducted in the absence of any commercial or financial relationships that could be construed as a potential conflict of interest.

## Footnotes

1 Other countries of origin include: El Salvador, Cuba, Guatemala, Puerto Rica, and Peru.
