# Supplementary material for: Changes in United States Latino/a High School Students’ Science Motivational Beliefs: Within Group Differences Across Science Subjects, Gender, Immigrant Status, and Perceived Support
Source: Front Psychol. 2019 Feb 22;10:380. doi: 10.3389/fpsyg.2019.00380 (PMC6395444; doi:10.3389/fpsyg.2019.00380)
Supplement: Supplementary file 1 [file Table_1.docx]

Supplemental Material 1. Items of Motivational Beliefs and Perceived Science Support Scale

Ability self-concept

- How good at [chemistry/physics/biology] are you? (1= *Not at all good*, 4= *Somewhat good*, 7= *Very good*)
- How good would you be at learning something new in [chemistry/physics/biology]? (1=*Not very good*, 4= *Somewhat good*, 7= *Very good*)
- Compared to other 9^th^/10^th^/11^th^ grade students, how good are you at [chemistry/physics/biology]? (1= *A lot worse*, 4= *About the same*, 7= *A lot better*)
- If you were to list all of the 9^th^/10^th^/11^th^ grade students from best to worst in [chemistry/physics/biology], where are you? (1= *One of the worst*, 4= *In the middle*, 7= *One of the best*)

Interest

- I find doing [chemistry/physics/biology]: (1= *Very boring*, 4= *Neither boring nor interesting*, 7= *Very interesting*)
- How much do you like [chemistry/physics/biology]? (1= *A little*, 4= *Somewhat*, 7= *A lot*)

Utility

- For me, being good in [chemistry/physics/biology] is: (1= *Not at all important*, 4= *Somewhat important*, 7= *Very important*)
- Compared to other subjects, how important is it to be good at [chemistry/physics/biology]? (1= *Not at all important*, 4= *Somewhat important*, 7= *Very important*)
- How useful is what you learn in [chemistry/physics/biology]? (1= *Not at all useful*, 7= *Very useful*)

Perceived science support: How often does your parent(s), older sibling, friends, or teacher…… (1 = *never,* 5 = *always*)

- Help enroll you in science lessons, workshops, or tutoring programs outside of class
- Tell you that you are good at science.
- Talk to you about how things are going in your science classes.
- Pressure you to do well in science.
- Talk about college majors and careers in science.
- Tell you how important doing well in science will be for your future.
- Look at science websites with you.
- Talk about news or current event related to science.
- Praise you for your school work in science.
- Help you feel better when science is hard.
- Like how you do things in science
- Say nice things about your grades in science.
- Like your study habit in science.
- Teach you about things you need to know.
- Teach you about things you want to know in science
