# Supplementary material for: Changes in United States Latino/a High School Students’ Science Motivational Beliefs: Within Group Differences Across Science Subjects, Gender, Immigrant Status, and Perceived Support
Source: Front Psychol. 2019 Feb 22;10:380. doi: 10.3389/fpsyg.2019.00380 (PMC6395444; doi:10.3389/fpsyg.2019.00380)
Supplement: Supplementary file 2 [file Table_2.docx]

Supplemental Material 2.

| Table S2. *Bivariate Correlations* | | | | | | | | | | | |  |  |  |  |  |  |  |  |  |  |  |  |  |  |  |  |  |  |  |
| --- | --- | --- | --- | --- | --- | --- | --- | --- | --- | --- | --- | --- | --- | --- | --- | --- | --- | --- | --- | --- | --- | --- | --- | --- | --- | --- | --- | --- | --- | --- |
|  |  | 1. | 2. | 3. | 4. | 5. | 6. | 7. | 8. | 9. | 10. | 11. | 12. | 13. | 14. | 15. | 16. | 17. | 18. | 19. | 20. | 21. | 22. | 23. | 24. | 25. | 26. | 27. | 28. | 29. |
| Motivational beliefs in 9th grade | | | | | |  |  |  |  |  |  |  |  |  |  |  |  |  |  |  |  |  |  |  |  |  |  |  |  |  |
| 1. | Biology ability self-concept | 1 |  |  |  |  |  |  |  |  |  |  |  |  |  |  |  |  |  |  |  |  |  |  |  |  |  |  |  |  |
| 2. | Biology interest | .63* | 1 |  |  |  |  |  |  |  |  |  |  |  |  |  |  |  |  |  |  |  |  |  |  |  |  |  |  |  |
| 3. | Biology utility | .36* | .60* | 1 |  |  |  |  |  |  |  |  |  |  |  |  |  |  |  |  |  |  |  |  |  |  |  |  |  |  |
| 4. | Chemistry ability self-concept | .49* | .37* | .30* | 1 |  |  |  |  |  |  |  |  |  |  |  |  |  |  |  |  |  |  |  |  |  |  |  |  |  |
| 5. | Chemistry interest | .29* | .44* | .54* | .68* | 1 |  |  |  |  |  |  |  |  |  |  |  |  |  |  |  |  |  |  |  |  |  |  |  |  |
| 6. | Chemistry utility | .30* | .43* | .70* | .48* | .62* | 1 |  |  |  |  |  |  |  |  |  |  |  |  |  |  |  |  |  |  |  |  |  |  |  |
| 7. | Physics ability self-concept | .46* | .30* | .31* | .68* | .52* | .26* | 1 |  |  |  |  |  |  |  |  |  |  |  |  |  |  |  |  |  |  |  |  |  |  |
| 8. | Physics interest | .27* | .40* | .50* | .39* | .59* | .49* | .63* | 1 |  |  |  |  |  |  |  |  |  |  |  |  |  |  |  |  |  |  |  |  |  |
| 9. | Physics utility | .16 | .32* | .65* | .30* | .53* | .71* | .47* | .67* | 1 |  |  |  |  |  |  |  |  |  |  |  |  |  |  |  |  |  |  |  |  |
| Motivational beliefs in 10th grade | | | | |  |  |  |  |  |  |  |  |  |  |  |  |  |  |  |  |  |  |  |  |  |  |  |  |  |  |
| 10. | Biology ability self-concept | .25* | .34* | .22* | .24* | .28* | .10 | .40* | .25* | .20 | 1 |  |  |  |  |  |  |  |  |  |  |  |  |  |  |  |  |  |  |  |
| 11. | Biology interest | .09 | .41* | .39* | .23* | .47* | .32* | .25* | .37* | .34* | .68* | 1 |  |  |  |  |  |  |  |  |  |  |  |  |  |  |  |  |  |  |
| 12. | Biology utility | .32* | .42* | .55* | .17 | .26* | .50* | .19 | .31* | .44* | .42* | .59* | 1 |  |  |  |  |  |  |  |  |  |  |  |  |  |  |  |  |  |
| 13. | Chemistry ability self-concept | .47* | .35* | .19 | .39* | .32* | .28* | .39* | .33* | .31* | .36* | .32* | .43* | 1 |  |  |  |  |  |  |  |  |  |  |  |  |  |  |  |  |
| 14. | Chemistry interest | .35* | .41* | .39* | .21* | .38* | .51* | .13 | .41* | .47* | .26* | .45* | .56* | .74* | 1 |  |  |  |  |  |  |  |  |  |  |  |  |  |  |  |
| 15. | Chemistry utility | .37* | .45* | .58* | .18 | .35* | .67* | .16 | .37* | .54* | .25* | .46* | .79* | .54* | .73* | 1 |  |  |  |  |  |  |  |  |  |  |  |  |  |  |
| 16. | Physics ability self-concept | .56* | .38* | .20 | .46* | .33* | .22* | .60* | .39* | .29* | .53* | .40* | .43* | .66* | .49* | .40* | 1 |  |  |  |  |  |  |  |  |  |  |  |  |  |
| 17. | Physics interest | .18 | .26* | .39* | .22* | .47* | .46* | .29* | .47* | .55* | .38* | .62* | .56* | .42* | .68* | .60* | .55* | 1 |  |  |  |  |  |  |  |  |  |  |  |  |
| 18. | Physics utility | .24* | .33* | .58* | .20 | .39* | .61* | .26* | .45* | .63* | .35* | .59* | .75* | .38* | .56* | .80* | .45* | .76* | 1 |  |  |  |  |  |  |  |  |  |  |  |
| Motivational beliefs in 11th grade | | | | |  |  |  |  |  |  |  |  |  |  |  |  |  |  |  |  |  |  |  |  |  |  |  |  |  |  |
| 19. | Biology ability self-concept | .37* | .27* | .20 | .16 | .08 | .12 | .24* | .12 | .08 | .48* | .41* | .46* | .36* | .33* | .36* | .38* | .23* | .35* | 1 |  |  |  |  |  |  |  |  |  |  |
| 20. | Biology interest | .17 | .41* | .41* | .26* | .43* | .37* | .23* | .27* | .30* | .43* | .75* | .60* | .32* | .47* | .52* | .32* | .53* | .59* | .64* | 1 |  |  |  |  |  |  |  |  |  |
| 21. | Biology utility | .24* | .34* | .44* | .09 | .22* | .45* | .02 | .18 | .30* | .20 | .46* | .70* | .26* | .49* | .66* | .23* | .44* | .65* | .53* | .69* | 1 |  |  |  |  |  |  |  |  |
| 22. | Chemistry ability self-concept | .29* | .15 | .05 | .22* | .07 | .10 | .28* | .22* | .10 | .27* | .11 | .26* | .52* | .36* | .25* | .48* | .26* | .22* | .50* | .28* | .23* | 1 |  |  |  |  |  |  |  |
| 23. | Chemistry interest | .20 | .29* | .27* | .23* | .33* | .35* | .22* | .33* | .28* | .17 | .37* | .39* | .40* | .52* | .44* | .38* | .47* | .42* | .41* | .53* | .49* | .71* | 1 |  |  |  |  |  |  |
| 24. | Chemistry utility | .23* | .33* | .43* | .13 | .29* | .47* | .14 | .36* | .37* | .26* | .42* | .56* | .29* | .50* | .63* | .29* | .47* | .64* | .44* | .56* | .69* | .52* | .74* | 1 |  |  |  |  |  |
| 25. | Physics ability self-concept | .37* | .16 | .23* | .30* | .19 | .11 | .49* | .28* | .19 | .47* | .37* | .33* | .39* | .27* | .26* | .56* | .47* | .41* | .54* | .41* | .28* | .60* | .44* | .39* | 1 |  |  |  |  |
| 26. | Physics interest | .22* | .17 | .29* | .25* | .41* | .39* | .39* | .42* | .42* | .38* | .49* | .40* | .29* | .44* | .40* | .43* | .41* | .60* | .32* | .48* | .41* | .38* | .43* | .46* | .70* | 1 |  |  |  |
| 27. | Physics utility | .20 | .18 | .45* | .16 | .37* | .45* | .30* | .46* | .53* | .29* | .41* | .50* | .27* | .43* | .55* | .31* | .69* | .73* | .34* | .46* | .56* | .37* | .45* | .68* | .54* | .74* | 1 |  |  |
| Predictors | |  |  |  |  |  |  |  |  |  |  |  |  |  |  |  |  |  |  |  |  |  |  |  |  |  |  |  |  |  |
| 28. | Female | .07 | .04 | .10 | -.07 | -.21* | -.15 | -.07 | -.12 | -.21* | -.12 | -.12 | .06 | -.11 | -.23* | -.04 | -.06 | .57* | -.06 | -.03 | -.07 | .09 | -.10 | -.06 | .04 | -.11 | -.24* | -.14 | 1 |  |
| 29. | Third generation | .01 | -.04 | -.18 | .12 | .10 | -.17 | .20* | .19 | -.05 | .36* | .17 | -.03 | .21* | .10 | -.10 | .25* | -.23* | -.08 | .15 | .08 | -.08 | .17 | .05 | -.05 | .27* | .19 | .11 | -.18 | 1 |
| 30. | Science support | .13 | .24* | .34* | .16 | .24* | .27* | .21* | .27* | .32* | .21* | .25* | .28* | .10 | .15 | .22* | .15 | .11 | .35* | .19 | .14 | .21 | .01 | .09 | .22* | .05 | .16 | .23* | -.07 | -.04 |
| *Note*. **p* <.05. | | | | | | | | |  |  |  |  |  |  |  |  |  |  |  |  |  |  |  |  |  |  |  |  |  |  |
