# Supplementary material for: Changes in United States Latino/a High School Students’ Science Motivational Beliefs: Within Group Differences Across Science Subjects, Gender, Immigrant Status, and Perceived Support
Source: Front Psychol. 2019 Feb 22;10:380. doi: 10.3389/fpsyg.2019.00380 (PMC6395444; doi:10.3389/fpsyg.2019.00380)
Supplement: Supplementary file 3 [file Table_3.docx]

Supplemental Material 3

| Table S3. *Perceived Science Support from Specific Source Predicting Science Motivational Beliefs* | | | | | | | | | | | | | |
| --- | --- | --- | --- | --- | --- | --- | --- | --- | --- | --- | --- | --- | --- |
|  |  | Science | | | Biology | | | Chemistry | | | Physics | | |
|  |  | Ability self-concept | Interest | Utility | Ability self-concept | Interest | Utility | Ability self-concept | Interest | Utility | Ability self-concept | Interest | Utility |
| Perceived support from parents | on intercept (S.E.) | .04  (.02) * | .03  (.02) | .06  (.02) ** | .06  (.02) ** | .05 (.02) * | .07  (.02) *** | .03  (.02) | .03 (.02) | .07  (.02) ** | .02  (.02) | .02  (.02) | .05  (.03) * |
|  | on slope (S.E.) | -.02 (.01) | -.02 (.01) | -.01 (.01) | -- | -.03 (.02) | -.00 (.01) | -.02 (.02) | -- | -.01 (.01) | -.03  (.01) * | -.03 (.01) | -.02 (.02) |
| Perceived support from sibling/cousins | on intercept (S.E.) | .04  (.01) ** | .04  (.02) * | .08  (.02) *** | .05  (.02) ** | .04 (.02) | .08  (.02) *** | .03  (.02) * | .05 (.02) * | .08  (.02) *** | .03  (.02) | .03  (.02) | .07  (.02) ** |
|  | on slope (S.E.) | .00  (.01) | -.01 (.01) | -.01 (.01) | -- | -.01 (.02) | -.02 (.01) | .01  (.01) | -- | -.00  (.01) | .01  (.01) | -.00 (.02) | -.01 (.01) |
| Perceived support from teachers | on intercept (S.E.) | .04  (.02) * | .06  (.02) ** | .07  (.02) *** | .04  (.02) * | .06 (.02) * | .07  (.02) *** | .04  (.02) * | .05 (.02) * | .07  (.02) ** | .04  (.02) * | .05  (.02) ** | .07  (.02) ** |
|  | on slope (S.E.) | -.02 (.01) | -.03 (.01) * | -.02 (.01) | -- | -.03 (.02) | -.02 (.01) | -.01 (.01) | -- | -.01  (.01) | -.03  (.01) * | -.03 (.02) | -.02 (.01) |
| Perceived support from friends | on intercept (S.E.) | .03  (.01) * | .04  (.02) | .07  (.03) * | .04  (.02) ** | .05 (.03) | .07  (.03) * | .03  (.02) | .04 (.03) | .07  (.03) * | .03  (.02) | .05  (.03) | .07  (.03) * |
|  | on slope (S.E.) | -.01 (.01) | -.02 (.01) | -.00 (.01) | -- | -.03 (.02) * | -.02 (.02) | .00  (.02) | -- | .01  (.02) | -.02  (.01) | -.02 (.02) | -.00 (.02) |
| *Note*. Four *separate* models (for each social agent) were run with the same set of covariates (not shown in this table): school (2 dummy variables), family income as covariates, being female, and immigrant generation status. S.E. = standard error. -- means linear growth curve has unsatisfactory model fit or non-significant variance, hence not followed up in this analysis. **p* < .05. ***p* < .01. ****p* <.001. | | | | | | | | | | | | | |
|  |  |  |  |  |  |  |  |  |  |  |  |  |  |
